# Auranofin mitigates systemic iron overload and induces ferroptosis via distinct mechanisms

**DOI:** 10.1038/s41392-020-00253-0

**Published:** 2020-07-31

**Authors:** Lei Yang, Hao Wang, Xiang Yang, Qian Wu, Peng An, Xi Jin, Weiwei Liu, Xin Huang, Yuzhu Li, Shiyu Yan, Shuying Shen, Tingbo Liang, Junxia Min, Fudi Wang

**Affiliations:** 1 Department of Nutrition, Precision Nutrition Innovation Center, School of Public Health, Zhengzhou University, 450001 Zhengzhou, China; 2 The First Affiliated Hospital, School of Public Health, Institute of Translational Medicine, Zhejiang University School of Medicine, 310058 Hangzhou, China; 3 Department of Nutrition and Health, Beijing Advanced Innovation Center for Food Nutrition and Human Health, China Agricultural University, 100193 Beijing, China; 4 School of Nursing, Xinxiang Medical University, 453003 Xinxiang, China

**Keywords:** Drug discovery, Gastrointestinal diseases

## Abstract

Iron homeostasis is essential for health; moreover, hepcidin-deficiency results in iron overload in both hereditary hemochromatosis and iron-loading anemia. Here, we identified iron modulators by functionally screening hepcidin agonists using a library of 640 FDA-approved drugs in human hepatic Huh7 cells. We validated the results in C57BL/6J mice and a mouse model of hemochromatosis (*Hfe*^*−/−*^ mice). Our screen revealed that the anti-rheumatoid arthritis drug auranofin (AUR) potently upregulates hepcidin expression. Interestingly, we found that canonical signaling pathways that regulate iron, including the Bmp/Smad and IL-6/Jak2/Stat3 pathways, play indispensable roles in mediating AUR’s effects. In addition, AUR induces IL-6 via the NF-κB pathway. In C57BL/6J mice, acute treatment with 5 mg/kg AUR activated hepatic IL-6/hepcidin signaling and decreased serum iron and transferrin saturation. Whereas chronically treating male *Hfe*^*−/−*^ mice with 5 mg/kg AUR activated hepatic IL-6/hepcidin signaling, decreasing systemic iron overload, but less effective in females. Further analyses revealed that estrogen reduced the ability of AUR to induce IL-6/hepcidin signaling in Huh7 cells, providing a mechanistic explanation for ineffectiveness of AUR in female *Hfe*^*−/−*^ mice. Notably, high-dose AUR (25 mg/kg) induces ferroptosis and causes lipid peroxidation through inhibition of thioredoxin reductase (TXNRD) activity. We demonstrate the ferroptosis inhibitor ferrostatin significantly protects liver toxicity induced by high-dose AUR without comprising its beneficial effect on iron metabolism. In conclusion, our findings provide compelling evidence that TXNRD is a key regulator of ferroptosis, and AUR is a novel activator of hepcidin and ferroptosis via distinct mechanisms, suggesting a promising approach for treating hemochromatosis and hepcidin-deficiency related disorders.

## Introduction

In most organisms, iron homeostasis is essential for survival. Under physiological conditions, the liver senses the body’s systemic iron content and—based on this iron content—produces the hormone hepcidin in real time in order to maintain iron homeostasis.^1^ Hepcidin restricts the uptake and recycling of iron by degrading ferroportin, the sole iron exporter.^2^ As the master iron-regulating hormone, hepcidin expression is tightly regulated by signaling pathways, including the BMP/SMAD pathway. BMP6 produced by endothelial cells regulates hepcidin expression and maintains systemic iron homeostasis via the protein hemojuvelin in hepatocytes.^3^ Iron-bound transferrin causes a conformational change in the transferrin receptors TFR1 and TFR2 at the surface of hepatocytes, causing a shift from TFR1/HFE complexes to TFR2/HFE complexes.^4,5^ In response to systemic iron loading, the BMP6/BMP receptor complex and/or the TFR2/HFE complex triggers the phosphorylation of SMAD1/5/8, which recruits SMAD4 in order to activate transcription of the *HAMP1* gene, which encodes hepcidin.^6,7^

In contrast, stress-related conditions such as the presence of inflammatory stimuli,^8^ erythropoietic factors,^9^ and hypoxia-inducing factors^1^ can alter *HAMP1* transcription via a variety of pathways. For example, chronic disease and microbial infection cause robust activation of the NF-κB pathway,^10^ and downstream pro-inflammatory cytokines such as IL-6^11^ and IL-1β^12^ regulate hepcidin expression via the Jak1/STAT3 pathway. In *IL-6* knockout mice, inflammatory stimuli do not induce hepcidin expression,^13^ suggesting that IL-6 plays a critical role in driving hepcidin expression. Interestingly, patients with chronic inflammation typically develop anemia due to increased hepcidin expression in response to increased inflammatory signaling.^1,8^ Moreover, hypoxia-induced erythropoietin (EPO) production can suppress hepcidin expression, thereby increasing iron absorption.^1,9^ EPO activates the STAT5 pathway, which produces erythroferrone, which in turn suppresses the BMP/SMAD/hepcidin axis,^9^ a process that also involves the MAPK/ERK signaling pathway.^14^

Given the central role that hepcidin plays in regulating iron homeostasis, changes in hepcidin expression are associated with a variety of iron-related diseases. For example, low hepcidin production is a major cause of haemochromatosis,^15^ whereas high hepcidin expression leads to iron-refractory iron deficient anemia (IRIDA).^16^ Low hepcidin expression has also been associated with severely impaired erythropoiesis in β-thalassemia^17^ with tissue iron deposition. Excess cellular iron leads to high levels of reactive oxygen species (ROS), which cause oxidative cell death and can lead to chronic complications.^15^ Interestingly, we previously reported that mouse models of iron overload develop ferroptosis-related liver damage.^18,19^

Ferroptosis is characterized as a lipid peroxidation‒induced, iron-dependent form of cell death and has been attributed to pathological tissue damage induced by ischemia/reperfusion and chemotherapeutic drugs.^20–23^ The antioxidant glutathione (GSH) is a robust scavenger of lipid peroxidation products, and impaired GSH metabolism is one of the major mechanisms underlying ferroptosis,^21,24^ including GSH deficiency (i.e., cystine/glutamate antiporter xc^−^ dysfunction)^25^ and impaired GSH utilization (i.e., by inhibiting glutathione peroxidase 4).^26^ In addition, thioredoxin is also a distinct thiol-containing antioxidant and can compensate for reduced GSH levels.^27^ Thioredoxin can react with ROS and is then recycled by thioredoxin reductase (TXNRD) enzymes.^28^ However, the role of the thioredoxin system in ferroptosis is poorly understood.

Given that both hepcidin and the synthetic peptide mini-hepcidin^29^ drastically reduce serum iron levels in animal models, several strategies have been used to identify novel compounds that might regulate hepcidin expression, thereby treating iron-related diseases. Although herbal extracts,^30–32^ epigenetic inhibitors,^33^ and sex hormones^34,35^ have all been reported to affect hepcidin expression and iron metabolism, none of these products are currently available for clinical use. Here, we found that the FDA-approved anti-rheumatoid arthritis (anti-RA) drug auranofin (AUR)^36^ potently upregulates hepcidin expression and induces ferroptosis both in vitro and in vivo.

## Results

### AUR is a potent inducer of hepcidin expression in vitro

**Fig. 1:**
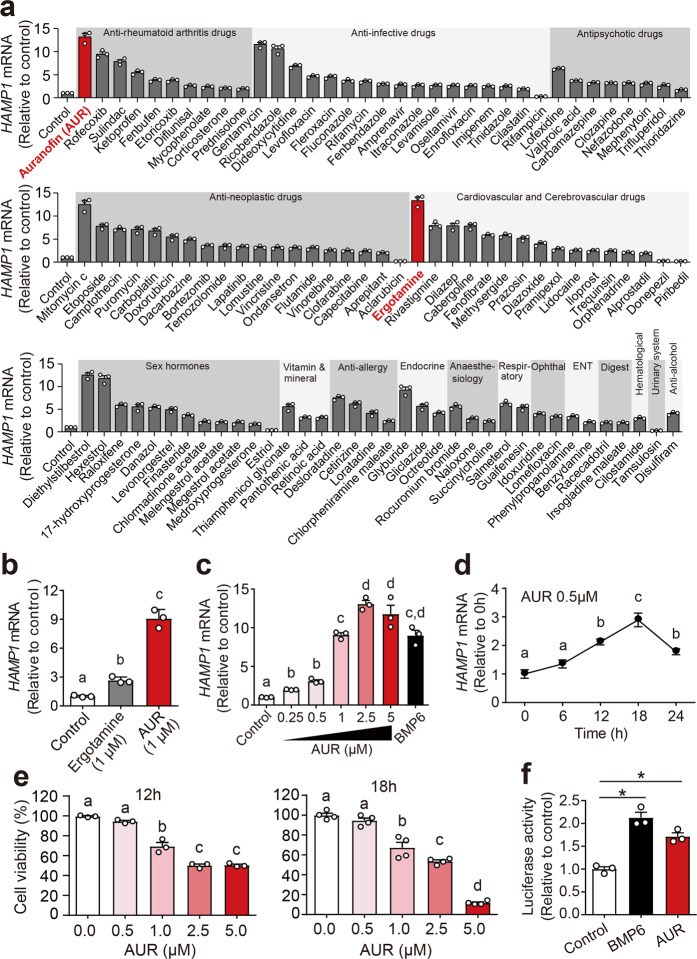
Auranofin potently upregulates hepcidin expression in Huh7 cells. **a***HAMP1* mRNA was measured in Huh7 cells treated for 12 h with 5 μM of the indicated drugs (see Table S1 for details); all 106 drugs caused a significant change in *HAMP1* mRNA compared to control-treated cells. **b***HAMP1* mRNA levels were measured in Huh7 cells treated with dihydro-ergotamine Mesylate (1 μM) or AUR (1 μM) for 12 h. **c***HAMP1* mRNA was measured in Huh7 cells treated for 12 h with the indicated concentration of AUR or BMP6(50 ng/ml). **d***HAMP1* mRNA was measured in Huh7 cells treated with 0.5 μM AUR for the indicated time. **e** Cell viability assay was performed in Huh7 cells treated with the indicated concentration of AUR for either 12 or 18 h. **f** Huh7 cells were co-transfected with pGL3-HAMP1 and the Renilla reporter construct; 36 h after transfection, the cells were treated with AUR (0.5 μM) or 50 ng/ml BMP6 for 18 h, after which luciferase activity was measured. The mRNA levels in panels **a**, **b** and **d** were normalized to *β-ACTIN* and are expressed relative to the mean control value. The cell line based in vitro experiments were repeated at least three independent times. Error bars indicate the SEM. The data in **a** and **e** were analyzed using the Student’s *t*-test (*p < 0.05). The data in **b**–**d** were analyzed using a one-way ANOVA with Tukey’s post hoc test; groups labeled without a common letter were significantly different (*p* < 0.05)

The BML-2843-0100 library containing 640 FDA-approved drugs was screened at a 5 μM concentration in Huh7 cells, and *HAMP1* mRNA was measured using RT-qPCR. As shown in Fig. 1a and Supplementary Table S1, a total of 100 drugs significantly upregulated hepcidin expression, and 6 drugs significantly downregulated hepcidin expression compared to control-treated cells (for details, see the extended results and discussion); based on their clinical applications, these 106 drugs were classified into 17 groups. Among the 100 drugs that upregulated hepcidin expression, both the anti-rheumatoid arthritis drug AUR and cardiovascular and cerebrovascular drug ergotamine induced the highest hepcidin expression in Huh7 cells (Fig. 1a). However, at a 1 μM low concentration, AUR treatment more potently upregulated hepcidin expression than ergotamine treatment (Fig. 1b). Specifically, in several case, patients treated with AUR developed anemia, and reducing or splitting the dose can alleviate these symptoms.^37–39^ These clinical observation indicated AUR’s potent roles in iron metabolism, but still lack further mechanistic study. We therefore focused on examining the effects of AUR. We then measured the time course and dose-response curve for AUR-induced upregulation of *HAMP1* mRNA levels (Fig. 1c, d). Notably, we found that treating Huh7 cells with high concentrations of AUR (≥1 μM) for 12 or 18 h significantly reduced cell viability measured using the CCK-8 assay (Fig. 1e). Thus, to minimize cell toxicity, we used 0.5 μM AUR in subsequent experiments. Consistent with selectively activating *HAMP1* expression, AUR also induced expression of a luciferase reporter construct driven by the *HAMP1* promoter (Fig. 1f).

### AUR upregulates hepcidin expression via the JAK2-STAT3 pathway

**Fig. 2:**
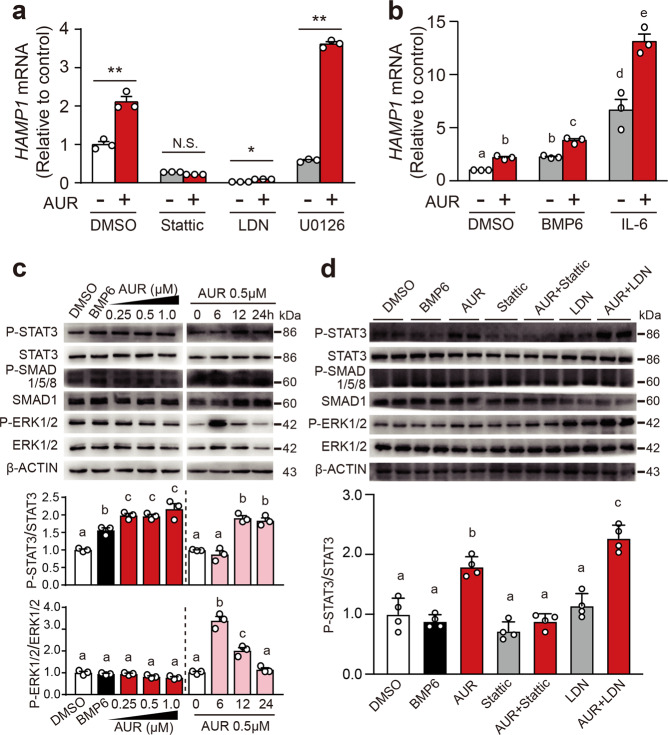
AUR upregulates hepcidin expression by activating the STAT3 pathway. **a***HAMP1* mRNA was measured in Huh7 cells pretreated with Stattic (10 μM), LDN-193189 (150 nM), or U0126 (10 μM) for 1 h, followed by an additional 18 h in the presence or absence of AUR (0.5 μM). **b***HAMP1* mRNA was measured in Huh7 cells treated with 50 ng/ml BMP6 or 50 ng/ml IL-6 in the presence or absence of AUR (0.5 μM) for 18 h. The mRNA levels were normalized to *β-ACTIN* and are expressed relative to the mean control value. **c** P-SMAD1/5/8, SMAD1, P-STAT3, STAT3, P-ERK1/2, ERK1/2, and β-ACTIN proteins were measured in untreated Huh7 cells (DMSO) and cells treated with BMP6 (50 ng/ml) or AUR for 12 h (left panel), or in Huh7 cells treated with 0.5 μM auranofin for the indicated times (right panel). **d** P-SMAD1/5/8, SMAD1, P-STAT3, STAT3, P-ERK1/2, ERK1/2, and β-ACTIN were measured in untreated Huh7 cells and cells treated with 50 ng/ml BMP6, 0.5 μM AUR, 10 μM Stattic, or 150 nM LDN-193189; where indicated, the cells were pretreated with Stattic or LDN, followed by AUR for an additional 18 h. For the protein quantification, P-STAT3/STAT3 and P-ERK1/2/ERK1/2 were normalized to their respective untreated groups. The cell line based experiments were repeated three independent times. Error bars indicate the SEM. The data in **a** were analyzed using the Student’s *t*-test (*p < 0.05, **p < 0.01, and N.S., not significant). The data in **b** and **d** were analyzed using a one-way ANOVA with Tukey’s post hoc test; groups labeled without a common letter were significantly different (*p* < 0.05)

Next, we examined whether the effect of AUR on *HAMP1* expression is mediated by canonical hepcidin-regulating pathways using pharmacological inhibitors. As shown in Fig. 2a, the ability of AUR to upregulate hepcidin expression in Huh7 cells was blocked by pretreating cells with either the BMP signaling inhibitor LDN193189 or the STAT3 inhibitor Stattic, but was significantly increased by the MEK1/2 inhibitor U0126. In contrast, co-treating cells with AUR and either BMP6 or IL-6 had a synergistic effect on inducing hepcidin expression (Fig. 2b). In addition, phospho-STAT3 levels were significantly increased by AUR treatment, reaching peak levels at 12 h (Fig. 2c), and pretreatment with Stattic reduced phospho-STAT3 and phospho-SMAD1/5/8 levels in AUR-treated Huh7 cells; in contrast, LDN193189 pretreatment suppressed phospho-SMAD1/5/8 levels but had no effect on phospho-STAT3 levels (Fig. 2d). Finally, neither Stattic nor LDN193189 affected phospho-ERK1/2 levels (Fig. 2d). Taken together, these data indicate that AUR activates the JAK2/STAT3 pathway but not the BMP/SMAD pathway.

### Acute treatment of AUR upregulates hepcidin expression and affects iron metabolism in mice

**Fig. 3:**
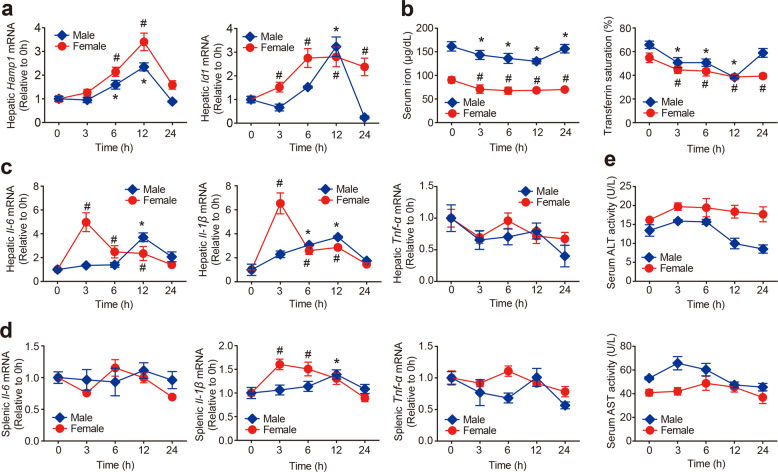
Acute AUR treatment increases hepcidin expression and affects iron metabolism in wild-type C57BL/6 J mice. Male and female wild-type C57BL/6 J mice (*n* = 8 mice per group) were given an intraperitoneal injection of AUR (5 mg/kg body weight). At the indicated times, hepatic *Hamp1* and *Id1* mRNA (**a**), serum iron content and transferrin saturation (**b**), hepatic *IL-6*, *IL-1β*, and *Tnf-α* mRNA (**c**), splenic *IL-6*, *IL-1β*, and *Tnf-α* mRNA (**d**), and serum ALT and AST activity (**e**) were measured. The mRNA levels were normalized to *β-actin* and are expressed relative to the mean value measured at 0 h. Error bars indicate the SEM. All data were analyzed using a one-way ANOVA with Tukey’s *post hoc* test; *p < 0.05 versus the respective 0-h value in the male group, and #*p* < 0.05 versus the respective 0-h value in the female group

Next, we examined whether AUR affects hepcidin expression in vivo by injecting C57BL/6 J mice with AUR and measuring hepatic *Hamp1* mRNA. As shown in Fig. 3a, AUR treatment upregulated hepatic hepcidin expression in both male and female mice, peaking at 12 h. Interestingly, AUR treatment also upregulated another downstream target of Smad4, *Id1*, again peaking at 12 h (Fig. 3a). In addition, AUR significantly reduced serum iron and transferrin saturation levels (Fig. 3b).

AUR has been shown previously to modulate NF-κB.^40^ Therefore, we examined the expression of pro-inflammatory cytokines in AUR-treated mice. We found that AUR increased hepatic *IL-6* mRNA, and hepatic and splenic *IL-1β* mRNA levels (Fig. 3c, d), generally peaking at 3 h; in contrast, hepatic and splenic *Tnf-α* expression were unaffected by AUR treatment. Interestingly, we found sex-specific differences with respect to the effect of AUR in changing hepatic hepcidin, serum iron, and hepatic and splenic *IL-6* and *IL-1β* levels (Fig. 3a–d). Lastly, AUR treatment did not cause liver toxicity, as serum ALT and AST levels were unchanged (Fig. 3e).

### AUR activates the IL-6/hepcidin axis via NF-κB

**Fig. 4:**
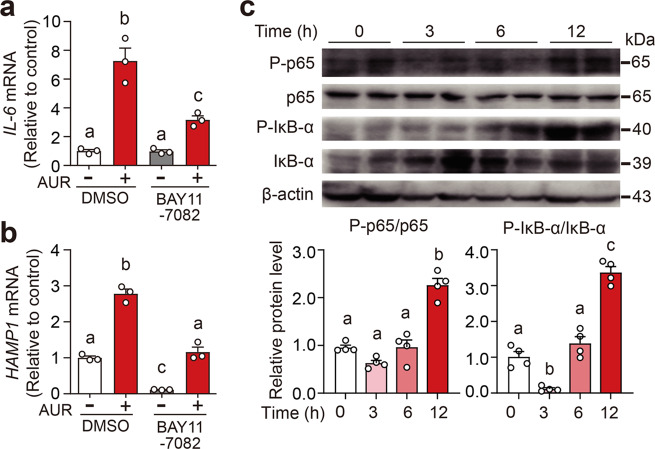
AUR activates the IL-6/hepcidin axis via the NF-кB pathway. **a**, **b***IL-6* and *HAMP1* mRNA were measured in Huh7 cells pretreated with 5 μM BAY11–7082 for 1 h, followed by 0.5 μM AUR for an additional 18 h. The mRNA levels were normalized to *β-ACTIN* and are expressed relative to the mean control value. **c** Hepatic P-p65, p65, P-IκB-α, IκB-α, and β-actin proteins were measured in male C57BL/6J mice at the indicated times after receiving an intraperitoneal injection of AUR (5 mg/kg body weight). In the summary graphs, P-p65 and P-IκB-α were normalized to p65 and IκB-α, respectively, and are expressed relative to the 0-h value. The experiments were repeated three independent times. Error bars indicate the SEM. All data were analyzed using a one-way ANOVA with Tukey’s *post hoc* test, and groups labeled without a common letter were significantly different (*p* < 0.05)

We found that AUR potently upregulates both *IL-6* and *HAMP1* mRNA levels In Huh7 cells, and this upregulation was significantly reduced when cells were pretreated with the NF-κB inhibitor BAY11–7082 (Fig. 4a, b), suggesting that NF-κB plays a critical role in mediating the effects of AUR. In addition, we found that AUR treatment increased the levels of hepatic phospho-p65 and phospho-IκB-α in male mice (Fig. 4c). Taken together, these data suggest that AUR induces hepcidin expression via the NF-κB/IL-6 pathway in both human liver cells and mice.

### Long-term treatment with 5 mg/kg AUR reduces iron burden in male *Hfe* knockout mice

**Fig. 5:**
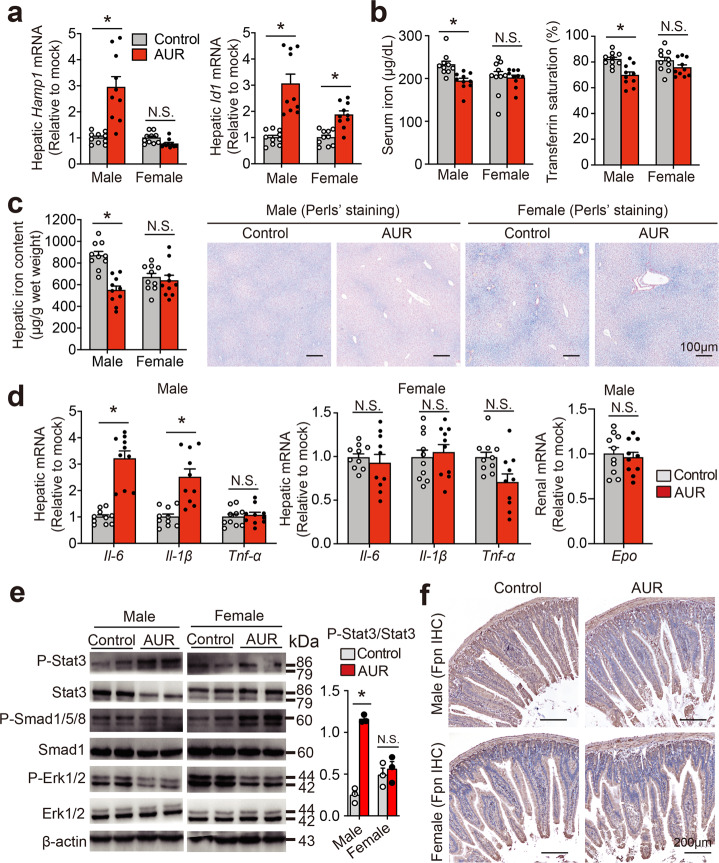
Long-term treatment with AUR reduces iron burden in male *Hfe*^−/−^ mice. Male and female 8-week-old *Hfe*^*−/−*^ mice (*n* = 10 mice per group) were given daily intraperitoneal injections of AUR (5 mg/kg) or vehicle (control) for 6 weeks, after which hepatic *Hamp1* and *Id1* mRNA (**a**), serum iron and transferrin saturation (**b**), hepatic iron content (**c**), Perls’ Prussian blue‒stained liver sections (**c**), hepatic *IL-6*, *Il-1β*, and *Tnf-α* mRNA (**d**), renal *Epo* mRNA (**d**), P-Stat3, Stat3, P-Smad1/5/8, Smad1, P-Erk1/2, Erk1/2, and β-actin protein (**e**), and duodenal ferroportin (Fpn) immunohistochemistry (**f**) were analyzed. The mRNA levels were normalized to *β-actin* and are expressed relative to the respective control group. In the summary plot in panel **e**, P-Stat3 protein was normalized to Stat3. Error bars indicate the SEM. *p < 0.05 and N.S., not significant (Student’s *t*-test)

Next, we measured the effect of AUR in *Hfe*^*−/−*^ mice, a classic model of hereditary hemochromatosis. Interestingly, we found that treating male *Hfe*^*−/−*^ mice with low-dose AUR (5 mg/kg body weight/day) increased hepatic hepcidin expression compared to control-treated mice, but had virtually no effect in female *Hfe*^*−/−*^ mice (Fig. 5a). Similarly, AUR reduced several iron parameters, including serum iron concentration, transferrin saturation, and hepatic iron, in male *Hfe*^*−/−*^ mice, but not female *Hfe*^*−/−*^ mice (Fig. 5b, c). This sex-specific effect was confirmed by staining liver sections with Perls’ Prussian blue, showing decreased iron in males but not in females (Fig. 5c). In addition, similar to our findings in wild-type C57BL/6 J mice, we found that AUR increased hepatic *IL-6* and *IL-1β* mRNA levels in male *Hfe*^*−/−*^ mice, but had no effect in female *Hfe*^*−/−*^ mice; in contrast, hepatic *Tnf-α* expression was unchanged in both male and female *Hfe*^*−/−*^ mice (Fig. 5d). Consistent with these results, we found that long-term AUR treatment also increased phospho-Stat3 levels in male *Hfe*^*−/−*^ mice, but had no effect in female *Hfe*^*−/−*^ mice (Fig. 5e). AUR treatment also significantly reduced both MCH (mean corpuscular hemoglobin) and MCHC (mean corpuscular hemoglobin concentration) in male *Hfe*^*−/−*^ mice, but had no significant effect in female mice; no other blood parameters were affected in either male or female *Hfe*^*−/−*^ mice (Supplementary Table S2). This AUR-induced reduction of MCH and MCHC in male *Hfe*^*−/−*^ mice was likely due to an upregulation of hepcidin expression, given that renal *Epo* mRNA levels were unchanged in AUR-treated male *Hfe*^*−/−*^ mice (Fig. 5d). We also found a decrease in Fpn (ferroportin) protein in the duodenum of AUR-treated male *Hfe*^*−/−*^ mice (Fig. 5f), suggesting decreased iron uptake.

Given our finding that AUR has sex-specific effects in both wild-type and *Hfe*^*−/−*^ mice, we examined whether sex hormones play a role in mediating AUR’s effects, particularly given the previous report that estrogen may be involved in regulating hepcidin.^41,42^ We therefore measured the effect of β-estradiol (E2) in Huh7 cells treated in the presence or absence of AUR. We found that treating cells with either 1 or 10 nM E2 significantly increased *IL-6* mRNA compared to control-treated cells, whereas higher concentrations of E2 (100 nM or 1 μM) had no effect (Supplementary Fig. S1a); moreover, E2 had no effect on *HAMP1* mRNA levels at any concentration tested (Supplementary Fig. S1b). Interestingly, even though 100 nM E2 had no effect on either IL-6 or hepcidin expression, this concentration of E2 significantly reduced the AUR-induced increase in both IL-6, phospho-STAT3 and hepcidin expression (Supplementary Fig. S1c-e), suggesting that E2 suppresses AUR-induced hepcidin expression via the IL-6/STAT3 axis. These data provide a possible explanation for our finding that AUR is less effective in female mice.

### High-dose AUR treatment induces hepatic ferroptosis in *Hfe*^*−/−*^ mice

**Fig. 6:**
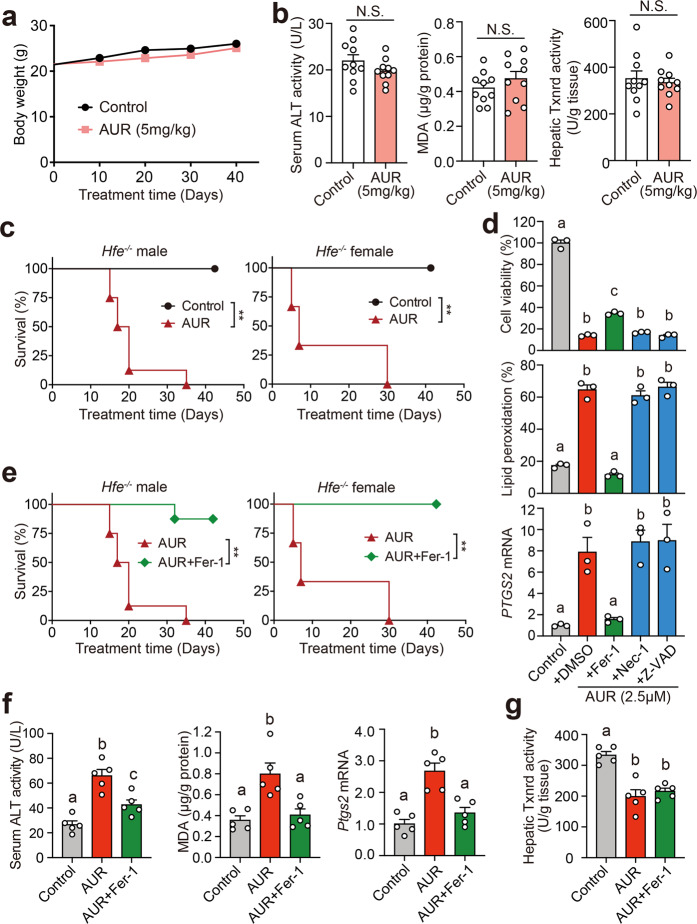
High-dose AUR induces ferroptosis and lethality in *Hfe*^*−/−*^ mice. **a**, **b** Body weight curve (**a**), serum ALT activity (**b**), hepatic MDA content (**b**) and hepatic Txnrd activity (**b**) was measured in male *Hfe*^*−/−*^ mice (*n* = 10 mice per group) receiving daily intraperitoneal injections of AUR (5 mg/kg) or saline (control) for 6 weeks starting at 8 weeks of age. **c** Kaplan–Meier survival curves of male and female *Hfe*^*−/−*^ mice (*n* = 10 mice per group) following daily intraperitoneal injections of saline (control) or AUR (25 mg/kg body weight) for 6 weeks starting at 16 weeks of age. **d** Huh7 cells were treated with 2.5 μM AUR and the indicated inhibitors; cell viability, lipid peroxidation, and *PTGS2* mRNA were measured 12 or 24 h after treatment. **e** Kaplan–Meier survival curves of male and female *Hfe*^*−/−*^ mice (*n* = 10 mice per group) treated daily with AUR (25 mg/kg body weight) either with or without Fer-1 (1 mg/kg body weight); for comparison purposes only, the AUR data are reproduced from panel **c**. **f**, **g** Serum ALT activity (**f**), hepatic MDA content (**f**), hepatic *Ptgs2* mRNA (**f**), and hepatic Txnrd activity (**g**) were measured in male *Hfe*^*−/−*^ mice (*n* = 5 mice per group) following daily intraperitoneal injections of saline (control), or AUR (25 mg/kg body weight) with or without Fer-1 (1 mg/kg body weight) for 6 weeks. The mRNA levels were normalized to *β-actin* and are expressed relative to the mean control value. Error bars indicate the SEM. The data in **a** and **b** were analyzed using the Student’s *t*-test and no significant difference was detected (N.S.). The cell line based experiments were repeated three independent times. The data in **c** and **e** were analyzed calculated using the log-rank (Mantel–Cox) test (**p < 0.01). All other summary data were analyzed using a one-way ANOVA with Tukey’s *post hoc* test; groups labeled without a common letter were significantly different (*p* < 0.05)

Previous studies reported AUR could be a potential chemotherapeutic drug for several tumor types.^43–45^ We next test the safety of AUR. We found that long-term 5 mg/kg AUR treatment in male *Hfe*^*−/−*^ mice did not alter the body weight or serum ALT compared with the control (Fig. 6a, b).

In contrast, when treated with high-dose AUR (25 mg/kg), *Hfe*^*−/−*^ mice showed 100% mortality within 42 days in both males and females (Fig. 6c). To identify the type of cell death underlying this toxic effect, we screened several inhibitors of various forms of cell death in Huh7 cells treated with high-dose AUR, including the apoptosis inhibitor Z-VAD-FMK (Z-VAD), the necroptosis inhibitor Necrostatin-1 (Nec-1), and the ferroptosis inhibitor Ferrostatin-1 (Fer-1). We found that Fer-1, but not Z-VAD or Nec-1, significantly increased the viability of AUR-treated Huh7 cells (Fig. 6d). In addition, we found that high-dose AUR increased several biomarkers of ferroptosis, including lipid peroxidation and *PTGS2* mRNA levels (Fig. 6d), and this increase was significantly reduced by Fer-1, but not Z-VAD or Nec-1, indicating that high-dose AUR selectively induces ferroptosis in hepatic cells.

To examine whether high-dose AUR induces ferroptosis in vivo, we treated male and female *Hfe*^*−/−*^ mice with AUR together with Fer-1. We found that Fer-1 could significantly rescue the mortality rate (Fig. 6e), serum ALT (Fig. 6f), hepatic malondialdehyde (MDA) content and *Ptgs2* mRNA (Fig. 6f) of *Hfe*^*−/−*^ mice treated with high-dose AUR, which indicates that targeting ferroptosis by Fer-1 could effectively protect against high-dose AUR induced hepatic toxicity. Notably, compared to the effects of low-dose AUR, high-dose AUR showed more robust effect on upregulation of hepatic *Hamp1* mRNA levels and decreasing levels of serum iron, transferrin saturation, and hepatic iron (Supplementary Fig. S2). Fer-1 treatment had no effect on AUR-regulated iron parameters (Supplementary Fig. S2), indicating that co-treatment with AUR and Fer-1 could prevent toxicity of high-dose AUR without compromising its beneficial effects on alleviating iron overload.

To explore potential underlying mechanisms of AUR-induced ferroptosis, we measured the well-known ferroptosis suppressors, including GSH and glutathione peroxidases (Gpx). We found that high-dose AUR did not alter hepatic GSH content, total Gpx activity and/or *Gpx4* expression (Supplementary Fig. S3). AUR has been shown to function as a pan-inhibitor of the thioredoxin reductase (TXNRD) family of enzymes, which serve as key regulators of various antioxidant pathways.^46^ We therefore tested whether AUR treatment affects hepatic TXNRD activity in *Hfe*^*−/−*^ mice and found that high-dose AUR significantly reduced total TXNRDs activity (Fig. 6g), whereas low-dose AUR had no effect (Fig. 6b). Moreover, Fer-1 had no effect on the high-dose AUR‒induced decrease in TXNRD activity (Fig. 6g). To further validate the regulatory roles of TXNRDs in ferroptosis, we treated wild-type C57BL/6J mice with TRi-1^44^(25 mg/kg), a TXNRD1 specific inhibitor for 2 weeks. As shown below, TRi-1 treatment resulted in body weight loss, an accumulation of hepatic lipid peroxidation (MDA content), increased *Ptgs2* mRNA level and serum ALT activity, which could be fully rescued by ferroptosis inhibitor ferrostatin-1(Supplementary fig. S4). These results suggest that inhibition of TXNRD1 could also induce ferroptosis in vivo.

## Discussion

Hepcidin, which serves as the master regulator of iron homeostasis, has emerged in recent years as an important target for treating iron-related diseases such as anemia and iron overload. Using functional screening, we previously identified several hepcidin expression antagonists and agonists present in Chinese herbs,^30^ black soybeans,^31^ and a library of small compounds. However, none of these compounds has been approved for clinical applications. Here, we screened a library of 640 FDA-approved drugs and identified AUR as a novel regulator of hepcidin expression both in vitro and in vivo. We also found that AUR has sex-specific effects with respect to alleviating iron overload in a mouse model of hereditary hemochromatosis. At the mechanistic level, we found that AUR has dual effect on iron metabolism and ferroptosis, shedding light on the feasibility of using AUR to treat iron overload‒related diseases.

AUR is approved for use in treating rheumatoid arthritis (RA) due to its anti-inflammatory activity.^47^ Based on our results from screening a total of 14 anti-RA drugs (10 of which significantly upregulated or downregulated hepcidin expression), we found that AUR, fenbufen, rofecoxib, sulindac, and ketoprofen robustly upregulated hepcidin expression in Huh7 cells (defined as a >5-fold increase in *HAMP1* mRNA compared to control-treated cells). Notably, AUR’s functions might vary in different cells. In AUR treated HepG2, we clearly observed declined *IL-6* mRNA level and no *HAMP1* mRNA response at 6 h compared with vesicle treated cells (Supplementary fig. S5), which was consistent with previous studies about AUR treatment in HepG2.^48^ Whereas in Huh7 cell line, AUR predominantly activates *IL-6* and *HAMP1* mRNA, which could be blocked by NF-κB inhibitor BAY11–7082 pretreatment. Consistent with Huh7, acute treatment with AUR in wild-type mice also induced a mild upregulation of IL-6 expression by activation of NF-κB in the liver, indicating AUR activating hepcidin via NF-κB/IL-6/STAT3 axis both in vitro and in vivo. Moreover, as anti-inflammation drug for RA, the effect of AUR on basal level has not been reported. With respect to its in vivo effects, we found that AUR had an unexpected mild pro-inflammatory effect (<10-fold increase in hepatic *IL-6* mRNA compared to control-treated mice) in both wild-type mice and *Hfe*^*−/−*^ mice. Thus, the distinct effects and mechanism of AUR on basal level or systematic inflammation need further investigation.

Sex is an important factor in iron metabolism. Males with *HFE* p.Cys282Tyr homozygous mutation show higher penetrance of hemochromatosis than their female counterparts.^15^ The female’ protective mechanisms against iron overload have been attributed to iron loss from female menstrual and sex hormones,^15^ whose roles in iron metabolism are not fully understood. Interestingly, we found that long-term AUR treatment had no effect in female *Hfe*^*−/−*^ mice. Moreover, and consistent with previous reports,^49^ we found that estrogen inhibited the AUR-induce increase in both *IL-6* and *HAMP1* mRNA levels in Huh7 cells. Together with our finding that AUR increases IL-6 expression in male mice but not in female mice, this indicates that estrogen suppresses hepcidin expression via an anti-inflammatory pathway. Previous studies found that estrogen regulates hepcidin expression either by directly binding to the estrogen response element in the *HAMP1* promoter^50^ or by regulating the GPR30/BMP6 axis.^42^ To our knowledge, this is the first study showing that estrogen has a previously unrecognized function in regulating both inflammation and hepcidin expression, suggesting AUR as a potential novel therapeutic strategy for male hemochromatosis.

AUR and other TXNRD inhibitors have been proposed as potential chemotherapeutic drugs for treating several types of cancer, including hepatocellular carcinoma,^45^ Hodgkin’s lymphoma,^43^ and head-and-neck cancer.^44^ In several cancer cell lines, AUR exhausts cellular thioredoxin, increasing ROS and ultimately causing oxidative cell death.^43–45^ Here, we found that AUR increases lipid peroxidation and *Ptgs2* mRNA levels in Huh7 cells, leading to cell death that was partially rescued by the ferroptosis-specific inhibitor Fer-1, but not inhibitors of other forms of cell death, implicating ferroptosis in AUR-mediated toxicity.

Ferroptosis is a recently identified iron-dependent and lipid peroxidation-dependent form of cell death. As reviewed by Stockwell et al., our group and others reported that ferroptosis serves as a major pathological mechanism in a wide range of organs, including the liver, heart, brain, and kidney.^20^ To the best of our knowledge, this is the first report that long-term treatment with AUR can lead to liver damage via ferroptosis in a dose-dependent manner. Interestingly, AUR triggered liver ferroptosis and 100% mortality by inhibiting TXNRD activity and increasing lipid peroxidation when given at a high dose (25 mg/kg body weight). However, a lower dose of AUR (5 to 10 mg/kg body weight) was not sufficient to induce ferroptosis (Fig. 6a, b) or mortality,^43–45^ and therefore might be relatively safe. In contrast, the potential toxic effect of high-dose AUR should be taken into consideration, and using a combination of ferroptosis inhibitors and AUR might be a safer strategy in clinic. Importantly, the TXNRD family of enzymes includes TXNRD1 and TXNRD2, which are expressed in the cytosol and mitochondria, respectively, and TXNRD3, which is expressed in the testes.^28^ Our findings provide experimental evidence supporting that both AUR (a pan-TXNRD inhibitor) and TRi-1 (a specific TXNRD1 inhibitor) could trigger lipid peroxidation and ferroptosis, indicating that targeting the thioredoxin system could serve as a novel strategy to modulate ferroptosis.

In summary, we report that the anti-RA drug auranofin has a dual function, increasing hepcidin expression via the NF-κB/IL-6/STAT3 signaling pathway and—at high doses—inducing ferroptosis by inhibiting the thioredoxin system (Supplementary Fig. S6). These findings provide compelling evidence that AUR may serve as a novel therapeutic strategy for treating hepcidin-deficiency related disorders, including hemochromatosis, particularly in male patients.

## Materials and methods

A detailed description of the materials and methods used in this study are available in the Supplementary Materials.

### Cell cultures and drug screening

Huh7 cells (a human hepatocarcinoma cell line) and human embryonic kidney (HEK293T) cells were obtained from the Cell Bank of Shanghai Institutes for Biological Sciences and cultured in Dulbecco’s modified Eagle’s medium supplemented with 10% fetal bovine serum (Gibco) and 1× penicillin–streptomycin (Gibco). The cells were cultured at 37 °C in 5% CO_2_. For drug screening, Huh7 cells were seeded in 6-well plates and treated for 12 h with the FDA-approved drug library (BML-2843–0100 National Compound Resource Center) at 5 μM, after which samples were collected and used for mRNA analysis. All cell line based in vitro experiments were repeated at least three independent times.

### Animal experiments

C57BL/6J mice (7–8 weeks of age, both males and females) were purchased from Vital River Laboratory Animal Technology Co., Ltd., Beijing, China. *Hfe*^*−/−*^ mice were kindly provided by Dr. Nancy C. Andrews. All mice were housed in a specific pathogen-free facility and fed an egg white‒based AIN-76A diet containing 50 mg/kg iron (Research Diets, Inc., New Brunswick, NJ). All mice were maintained under a 12-h/12-h light/dark cycle, and all animal experiments were approved by the Institutional Animal Care and Use Committee of Zhengzhou University and Zhejiang University.

### Statistical analysis

All summary data are expressed as the mean ± SEM. To meet the assumption of homogeneity of variance, an analysis of variance (ANOVA) was performed, followed by Tukey’s multiple comparison test. The Student’s *t*-test was used to compare the difference between two groups, and differences were considered statistically significant at *p* < 0.05.

## Supplementary information

Supplementary Material
