# Supplementary material for: Auranofin mitigates systemic iron overload and induces ferroptosis via distinct mechanisms
Source: Signal Transduct Target Ther. 2020 Jul 31;5:138. doi: 10.1038/s41392-020-00253-0 (PMC7393508; doi:10.1038/s41392-020-00253-0)
Supplement: Supplementary file 1 — Supplementary Material [file 41392_2020_253_MOESM1_ESM.docx]

**Supplementary Material**

**Auranofin mitigates systemic iron overload and induces ferroptosis via distinct mechanisms**

Lei Yang, Hao Wang, Xiang Yang, Qian Wu, Peng An, Xi Jin, Weiwei Liu, Xin Huang, Yuzhu Li，Shiyu Yan, Shuying Shen, Tingbo Liang, Junxia Min, and Fudi Wang

**This file includes:**

Extended Results and Discussion

Extended Methods

Supplementary Tables S1-S3

Supplementary Figures S1-S6

**EXTENDED RESULTS AND DISCUSSION**

Among the 100 drugs that significantly increased hepcidin expression (Figure 1A-C and Table S1), nine—hexestrol, diethylstilbestrol, glyburide, rofecoxib, gentamycin sulfate, ergotamine, ricobendazole, mitomycin c, and auranofin (AUR) increased hepcidin expression by >9-fold compared to control. Hexestrol and Diethylstilbestrol are synthetic non-steroidal estrogens, and exposure to estrogens has long been recognized as a risk factor for developing a variety of cancers^1^. Moreover, diethylstilbestrol is associated with severe side effects, including veno–thromboembolic events (VTEs), heart failure, stroke, and gynecomastia, which limits its clinical application^2^. Glyburide, an orally administered, long-acting sulfonylurea-based hypoglycemic agent, can cause severe hypoglycemia that can be fatal if given at the wrong dose^3^. Nonsteroidal anti-inflammatory drugs (NSAIDs), including rofecoxib, celecoxib, etoricoxib, lumiracoxib, and valdecoxib, were found to increase the risk of major vascular events such as non-fatal myocardial infarction, non-fatal stroke, and vascular-related death ^4^. Aminoglycosides such as gentamycin are potent antibiotics; however, the therapeutic feasibility of gentamycin is limited due to severe toxicity, including irreversible hearing loss in ∼20% of patients^5^. Ergotamine was is used as an abortifacient (i.e., to induce abortion) and to control postpartum bleeding, and chronic ergotamine intake has been associated with angiospastic occlusion of the superficial femoral artery and fibrous dysplasia of the blood vessels and heart valves^6,7^. Bendazoles, including ricobendazole, reduce the viability of protoscolices and tapeworm cysts, and its hepatic metabolite albendazole sulfoxide is also active against larval cestodes. Although teratogenicity of benzimidazoles has not been extensively studied, all benzimidazoles should be avoided—where possible—in women of childbearing age^8^. The most common toxic side effects associated with mitomycin including anorexia, vomiting, and myelosuppression; given that these adverse effects are difficult to predictable and are associated with a high rate of morbidity and mortality, the use of this agent as an adjuvant therapy appears unwarranted^9^.

Auranofin, a drug used to treat rheumatoid arthritis^10^, has also being investigated for possible therapeutic applications in a number of other diseases and conditions, including cancer, neurodegenerative disorders, HIV/AIDS, parasitic infections, and bacterial infections^11,12^. Importantly, auranofin has rather modest adverse effects, most of which are associated with long-term use for treating chronic disease and can include gastrointestinal complaints, which can develop in the first few months of treatment. Specifically, approximately 40% of patients develop loose stools^13^, and several case reports showed anemia without further mechanistic investigation^14-16^, indicating its potent roles in iron metabolism. In most cases, reducing or splitting the dose can alleviate these symptoms^13^.

**EXTENDED MATERIALS AND METHODS**

**Cell Counting Kit-8 (CCK-8) assay**

The Cell Counting Kit-8 (Dojindo, Japan) was used in accordance with the manufacturer’s instructions to measure auranofin-induced cytotoxicity. Cells (10^4^cells/well) were seeded into 96-well plates and cultured for 24 h. The cells were then incubated with vehicle (DMSO, 0.1% v/v in culture media) or 0, 0.5, 1, 2.5, or 5 μM auranofin (Sigma) for an additional 12 or 18 hours. For the CCK-8 assay, 10 μl of CCK-8 solution was added to each well and the cells were incubated at 37°C for 1.5 hours. The absorbance at 450 nm (OD_450_) of the cells was then measured using a microplate reader (Molecular Devices). Cell viability was calculated as a percentage relative to the DMSO group.

**Luciferase reporter assay**

The luciferase reporter assay was performed in accordance with the manufacturer's instructions (Dual-Luciferase Reporter Assay System; Promega). HEK293T cells grown in 24-well plates (10^5^ cells/well) were transiently co-transfected with 0.49 μg/well of the reporter plasmid pGL3-HAMP1, which contains the 2.7-kb 5’-flanking genomic region of the human *HAMP1* gene and the 5’-UTR (from −2700 bp to +71 bp), and 10 ng/well Renilla luciferase plasmid in accordance with the manufacturer's instructions (Lipofectamine 2000; Invitrogen). Thirty-six hours after transfection, the cells were exposed to either DMSO (0.1% v/v) or auranofin (0.5μm) and cultured for an additional 18 hours. Normalized promoter activity was measured relative to Renilla luminescence as described previously^17,18^.

**Animal experiments**

Mice (male and female) of the indicated strains were randomly assigned to the control and test groups, each group contained 5–10 mice. C57BL/6J mice were given a single intraperitoneal injection of vehicle (0.1% DMSO in saline) or auranofin (5 mg/kg body weight) and then sacrificed 0, 3, 6, 12, or 24 hours post-injection. C57BL/6J mice were received daily intraperitoneal injections of vehicle (0.1% DMSO in saline) or TRi-1 (25 mg/kg body weight), either with or without ferrostatin-1 (1 mg/kg body weight) for 2 weeks. *Hfe^-/-^*mice^19^ received daily intraperitoneal injections of vehicle (0.1% DMSO in saline), low-dose auranofin (5 mg/kg body weight), or high-dose auranofin (25 mg/kg body weight) either with or without ferrostatin-1 (1 mg/kg body weight) for 6 weeks, after which they were sacrificed, and tissue samples were collected for analysis.

**Liver damage and fibrosis**

Serum alanine aminotransferase (ALT) was measured using an enzymatic assay kit (Shensuoyoufu, China). Liver sections were stained with Sirius red to measure fibrosis.

**Lipid peroxidation**

*In vitro* cellular lipid peroxidation was measured using FACS analysis with C11-BODIPY (581/591) (Invitrogen) staining. *In vivo* tissue lipid peroxidation was measured by measuring malondialdehyde (MDA) content using an MDA assay kit (cat. #BC0025, Solarbio, China).

**Thioredoxin reductase activity**

Thioredoxin reductase (TXNRD) activity in liver tissue was measured using an enzymatic assay kit (cat. #BC1155, Solarbio, China).

**Glutathione and Glutathione peroxidases activity**

Glutathione (GSH) content and glutathione peroxidases (Gpx) activity in the liver tissues were measured by using GSH assay kit (cat. #BC1175, Solarbio, China) and total Gpxs enzymatic assay kit (cat. #S0056, Beyotime, China).

**RNA extraction and quantitative RT-PCR**

Total RNA was extracted as described previously ^20^ using the SuperfecTRI Total RNA Isolation Regent (Pufei, Shanghai, China), and cDNA was synthesized using the PrimerScript RT reagent kit with gDNA Eraser (cat. #R047; Takara, Beijing, China). mRNA transcripts were amplified using the primers listed in Supplementary Table S3, and the relative expression of each target gene was normalized to the respective *β-actin* mRNA level.

**Western blot analysis**

Cultured cells and mouse tissues were lysed in accordance with the manufacturer's instructions (Nanjing KeyGEN Biotech, Nanjing, China). Protein concentration was measured using a BCA protein assay (Beyotime Biotech).

The following primary antibodies were used: phospho-Smad1/5/8 (1:1000; Cell Signaling #9511S), phospho-Smad1/5 (1:1000; Cell Signaling #9516S), Smad1 (1:1000; Cell Signaling #9743S), phospho-Stat3 (1:1000; Cell Signaling #9145S), Stat3 (1:1000; Cell Signaling #4904T), phospho-Erk1/2 (1:1000; Cell Signaling #4376S), Erk1/2 (1:1000; Cell Signaling #4695T), phospho-P65 (1:1000; Cell Signaling #3033S), p65 (1:1000; Cell Signaling #8242S), phospho-IκBα (1:1000; Cell Signaling #9246S), IκBα (1:1000; Cell Signaling #9242S), and β-actin (1:2000; Santa Cruz #sc-47778).

**Compounds and reagents**

Auranofin (#HY-B1123, MCE, USA), TRi-1 (#T5481, Topscience, China), Ferrostatin-1 (#T6500, Topscience, China), Dihydro-ergotamine mesylate (#5751, Selleck, China), Recombinant Human IL-6 Protein (#206-IL-010, R&D Systems, USA), Recombinant Human BMP-6 Protein (#507-BP-020, R&D Systems, USA), Stattic (#S7947, Sigma, USA), LDN-193189 (#S2618, Selleck, USA), U0126 (#S1102, Selleck, USA), BAY11-7082 (#HY-13453, MCE, USA), Necrostatin-1 (#S8037, Selleck, USA), Z-VAD (#S7023, Selleck, USA), β-estradiol (#E2758, Sigma, USA).

**Immunohistochemistry**

Immunohistochemistry (IHC) was used to detect Fpn as previously described^20^. The dissected murine tissues were fixed in Bouins’ Solution (Sigma) and embedded in paraffin. Serial sections (5 μm) were deparaffinized, blocked (5% normal goal serum and 1% BSA in PBS) and then incubated overnight with a rabbit anti-ferroportin primary antibody at 1:200 dilution at 4°C under humidified conditions, followed by incubation for 1 hour with goat anti-rabbit secondary antibody (1:500 dilution, Proteintech Group, Inc) at room temperature.

**Serum iron and tissue non-heme iron measurements**

Serum iron, transferrin saturation, and tissue non-heme iron were measured as described previously^20^. Serum iron concentrations were determined using a serum iron-unsaturated iron binding capacity kit (Thermo DMA) according to the manufacturer’s instructions. The quantification data of tissue non-heme iron are presented as micrograms of iron per gram wet weight of tissue.

**References**

1. Cavalieri, E. & Rogan, E. The molecular etiology and prevention of estrogen-initiated cancers. *Molecular Aspects of Medicine* **36,** 1–55 (2014).

2. Shamash, J. *et al.* A multi-centre randomised phase III trial of Dexamethasone vs Dexamethasone and diethylstilbestrol in castration-resistant prostate cancer: immediate vs deferred Diethylstilbestrol. *Br. J. Cancer* **104,** 620–628 (2011).

3. Lim, C. C. T. *et al.* Severe Hypoglycemia Associated with an Illegal Sexual Enhancement Product Adulterated with Glibenclamide: MR Imaging Findings. *Radiology* **250,** 193–201 (2009).

4. Grosser, T., Ricciotti, E. & FitzGerald, G. A. The Cardiovascular Pharmacology of Nonsteroidal Anti-Inflammatory Drugs. *Trends in Pharmacological Sciences* **38,** 733–748 (2017).

5. Matt, T. *et al.* Dissociation of antibacterial activity and aminoglycoside ototoxicity in the 4-monosubstituted 2-deoxystreptamine apramycin. *Proceedings of the National Academy of Sciences* **109,** 10984–10989 (2012).

6. Egermayer, P. Epidemics of vascular toxicity and pulmonary hypertension: what can be learned? *J Intern Med* **247,** 11–17 (2000).

7. Redfield, M. M. Valve Disease Associated with Ergot Alkaloid Use: Echocardiographic and Pathologic Correlations. *Ann Intern Med* **117,** 50 (1992).

8. Liu, L. X. & Weller, P. F. Antiparasitic drugs. *N Engl J Med* **334,** 1178–1184 (1996).

9. Doll, D. C., Weiss, R. B. & Issell, B. F. Mitomycin: ten years after approval for marketing. *JCO* **3,** 276–286 (1985).

10. Tepperman, K. *et al.* Intestinal uptake and metabolism of auranofin, a new oral gold-based antiarthritis drug. *Science* **225,** 430–432 (1984).

11. Celegato, M. *et al.* Preclinical activity of the repurposed drug auranofin in classical Hodgkin lymphoma. *Blood* **126,** 1394–1397 (2015).

12. Tejman-Yarden, N. *et al.* A Reprofiled Drug, Auranofin, Is Effective against Metronidazole-Resistant Giardia lamblia. *Antimicrob. Agents Chemother.* **57,** 2029–2035 (2013).

13. Kean, W. F. & Kean, I. R. L. Clinical pharmacology of gold. *Inflammopharmacol* **16,** 112–125 (2008).

14. Giannini, E. H., Brewer, E. J. & Person, D. A. Auranofin in the treatment of juvenile rheumatoid arthritis. *J. Pediatr.* **102,** 138–141 (1983).

15. Brewer, E. J., Giannini, E. H. & Person, D. A. Early experiences with auranofin in juvenile rheumatoid arthritis. *Am. J. Med.* **75,** 152–156 (1983).

16. Williame, L. M., Joos, R., Proot, F. & Immesoete, C. Gold-induced aplastic anemia. *Clin. Rheumatol.* **6,** 600–605 (1987).

17. Casanovas, G., Mleczko-Sanecka, K., Altamura, S., Hentze, M. W. & Muckenthaler, M. U. Bone morphogenetic protein (BMP)-responsive elements located in the proximal and distal hepcidin promoter are critical for its response to HJV/BMP/SMAD. *J. Mol. Med.* **87,** 471–480 (2009).

18. Pandur, E. *et al.* Prohepcidin binds to the HAMPpromoter and autoregulates its own expression. *The Biochemical journal* **451,** 301–311 (2013).

19. Nicolas, G. *et al.* Constitutive hepcidin expression prevents iron overload in a mouse model of hemochromatosis. *Nat Genet* **34,** 97–101 (2003).

20. Zhang, Z. *et al.* Ferroportin1 deficiency in mouse macrophages impairs iron homeostasis and inflammatory responses. *Blood* **118,** 1912–1922 (2011).

**Supplementary Tables**

**Supplementary Table S1.** List of 106 drugs that significantly upregulated/downregulated *HAMP1* expression in Huh7 cells treated with 5μM of each drug

| **Antineoplastic drugs (19)** | **Fold change of *HAMP1***  **（Relative to each untreated control）** |
| --- | --- |
| Aclarubicin | 0.21↓ |
| Camptothecin | 7.17↑ |
| Etoposide | 7.82↑ |
| Mitomycin c | 12.52↑ |
| Puromycin 2HCl | 7.11↑ |
| Doxorubicin HCl | 5.48↑ |
| Lapatinib | 3.36↑ |
| Bortezomib | 3.59↑ |
| Clofarabine | 2.48↑ |
| Lomustine | 3.25↑ |
| Vincristine sulfate | 3.16↑ |
| Vinorelbine | 2.57↑ |
| Carboplatin | 6.80↑ |
| Dacarbazine | 4.88↑ |
| Flutamide | 3.08↑ |
| Temozolomide | 3.48↑ |
| Ondansetron | 3.12↑ |
| Aprepitant | 2.02↑ |
| Capecitabine | 2.31↑ |

| **Cardiovascular and Cerebrovascular drugs (16)** | **Fold change of *HAMP1***  **（Relative to each untreated control）** |
| --- | --- |
| Donepezil HCl | 0.26↓ |
| Lidocaine | 2.54↑ |
| Prazosin HCl | 5.17↑ |
| Piribedil HCl | 0.19↓ |
| Ergotamine D-tartrate | 13.38↑ |
| Rivastigmine | 7.99↑ |
| Diazoxide | 3.99↑ |
| Iloprost | 2.46↑ |
| Cabergoline | 7.85↑ |
| Dilazep | 7.91↑ |
| Pramipexol | 2.84↑ |
| Fenofibrate | 5.84↑ |
| Trequinsin HCl | 2.37↑ |
| Methysergide | 5.79↑ |
| Alprostadil | 1.90↑ |
| Orphenadrine citrate | 2.09↑ |

| **Antipsychotic drugs (8)** | **Fold change of *HAMP1***  **（Relative to each untreated control）** |
| --- | --- |
| Clozapine | 3.20↑ |
| Mephenytoin | 3.05↑ |
| Valproic acid | 3.69↑ |
| Lofexidine | 6.30↑ |
| Carbamazepine | 3.23↑ |
| Nefazodone | 3.17↑ |
| Thioridazine HCl | 1.67↑ |
| Trifluperidol 2HCl | 2.60↑ |

| **Anti-infective drugs（17）** | **Antibiotic drugs (9)** | **Fold change of *HAMP1***  **（Relative to each untreated control）** |
| --- | --- | --- |
|  | Imipenem | 2.48↑ |
|  | Enrofloxacin | 2.54↑ |
|  | Fluconazole | 3.69↑ |
|  | Fleroxacin | 4.54↑ |
|  | Gentamycin sulfate | 11.59↑ |
|  | Itraconazole | 2.66↑ |
|  | Levofloxacin HCl | 4.65↑ |
|  | Tinidazole | 2.43↑ |
|  | Cilastatin | 1.90↑ |
|  | **Antiviral drugs（3）** |  |
|  | Oseltamivir | 2.64↑ |
|  | 2',3' - dideoxycytidine | 6.87↑ |
|  | Amprenavir | 2.82↑ |
|  | **Anti-tuberculosis drugs（2）** |  |
|  | Rifampicin | 0.28↓ |
|  | Rifamycin sv | 3.57↑ |
|  | **Antiparasitic drugs（3）** |  |
|  | Ricobendazole | 10.64↑ |
|  | Fenbendazole | 3.00↑ |
|  | Levamisole HCl | 2.66↑ |

| **Anti-rheumatoid arthritis drugs（10）** | **DMARD (1)** | **Fold change of *HAMP1***  **（Relative to each untreated control）** |
| --- | --- | --- |
|  | Auranofin | 13.2↑ |
|  | **NSAIDs（6）** |  |
|  | Rofecoxib | 9.47↑ |
|  | Sulindac | 7.87↑ |
|  | Etoricoxib | 3.75↑ |
|  | Diflunisal | 2.61↑ |
|  | Fenbufen | 3.80↑ |
|  | Ketoprofen+D153 | 5.48↑ |
|  | **Hormone drugs（2）** |  |
|  | Corticosterone | 2.10↑ |
|  | Prednisolone | 1.95↑ |
|  | **Immunosuppressors（1）** |  |
|  | Mycophenolate mofetil | 2.37↑ |

| **Sex hormones (12)** | **Fold change of *HAMP1***  **（Relative to each untreated control）** |
| --- | --- |
| Raloxifene HCl | 5.92↑ |
| Chlormadinone acetate | 2.22↑ |
| Danazol | 5.45↑ |
| Diethylstilbestrol | 12.58↑ |
| Estriol | 0.35↓ |
| Finasteride | 3.64↑ |
| Hexestrol | 11.80↑ |
| 17- hydroxyprogesterone | 5.58↑ |
| Levonorgestrel | 4.93↑ |
| Medroxyprogesterone 17-acetate | 1.70↑ |
| Megestrol acetate | 2.04↑ |
| Melengestrol acetate | 2.13↑ |

| **Vitamin and minerals（3）** | **Fold change of *HAMP1***  **（Relative to each untreated control）** |
| --- | --- |
| Retinoic acid | 3.16↑ |
| Pantothenic acid | 3.17↑ |
| Thiamphenicol glycinate | 5.59↑ |

| **Anti-allergy drugs（4）** | **Fold change of *HAMP1***  **（Relative to each untreated control）** |
| --- | --- |
| Cetirizine 2HCl | 6.19↑ |
| Chlorpheniramine maleate | 2.37↑ |
| Desloratadine | 7.52↑ |
| Loratadine | 4.14↑ |

| **Endocrine system drugs（3）** | **Fold change of *HAMP1***  **（Relative to each untreated control）** |
| --- | --- |
| Octreotide | 4.16↑ |
| Glyburide | 9.29↑ |
| Gliclazide | 5.68↑ |

| **Anesthesiology drugs（3）** | **Fold change of *HAMP1***  **（Relative to each untreated control）** |
| --- | --- |
| Rocuronium bromide | 5.58↑ |
| Naloxone HCl | 2.89↑ |
| Succinylcholine | 2.36↑ |

| **Respiratory drugs（2）** | **Fold change of *HAMP1***  **（Relative to each untreated control）** |
| --- | --- |
| Salmeterol | 6.27↑ |
| Guaifenesin | 5.40↑ |

| **Ophthalmological drugs（2）** | **Fold change of *HAMP1***  **（Relative to each untreated control）** |
| --- | --- |
| Idoxuridine | 3.96↑ |
| Lomefloxacin HCl | 3.39↑ |
| **ENT medicines（2）** |  |
| Benzydamine | 2.13↑ |
| Phenylpropanolamine | 3.38↑ |

| **Digestive system agents（2）** | **Fold change of *HAMP1***  **（Relative to each untreated control）** |
| --- | --- |
| Racecadotril | 2.05↑ |
| Irsogladine maleate | 2.02↑ |

| **Hematological system drugs（1）** | **Fold change of *HAMP1***  **（Relative to each untreated control）** |
| --- | --- |
| Cilostamide | 3.01↑ |

| **Urinary system drugs（1）** | **Fold change of *HAMP1***  **（Relative to each untreated control）** |
| --- | --- |
| Tamsulosin HCl | 0.28↓ |

| **Anti-alcohol drugs（1）** | **Fold change of *HAMP1***  **（Relative to each untreated control）** |
| --- | --- |
| Disulfiram | 4.07↑ |

Abbreviations: DMARD, disease-modifying antirheumatic drug; ENT, ear, nose, and throat; NSAIDs, nonsteroidal anti-inflammatory drugs. **↓** downregulation and ↑ upregulation.

**Supplementary Table S2.** Blood parameters in male and female *Hfe^-/-^* mice treated with low-dose AUR (5 mg/kg body weight)

Male *Hfe^-/-^* mice (n=10 mice per group)

|  | **Control** | **Auranofin** | ***p*-value^a^** |
| --- | --- | --- | --- |
| RBC (10^12^/L) | 7.73±0.19 | 7.67±0.70 | 0.87 |
| Hb (g/dL) | 14.02±0.42 | 13.66±1.04 | 0.46 |
| HCT (%) | 37.37±1.13 | 38.66±3.39 | 0.40 |
| MCV (fL) | 49.68±1.60 | 49.62±0.42 | 0.92 |
| MCH (pg) | 18.65±0.74 | 17.5±0.44 | 0.02 |
| MCHC (g/dL) | 37.53±0.80 | 35.3±0.70 | 0.0004 |
| PLT (10^9^/L) | 623.67±84.76 | 546.2±18.19 | 0.08 |

Female *Hfe^-/-^* mice (n=10 mice per group)

|  | **Control** | **Auranofin** | ***p‒*-value^a^** |
| --- | --- | --- | --- |
| RBC (10^12^/L) | 6.97±0.42 | 7.03±0.32 | 0.81 |
| Hb (g/dL) | 13.48±0.77 | 13.98±0.69 | 0.34 |
| HCT (%) | 35.18±2.23 | 36.25±1.40 | 0.42 |
| MCV (fL) | 50.57±2.10 | 51.86±0.86 | 0.23 |
| MCH (pg) | 19.37±0.63 | 19.9±0.29 | 0.12 |
| MCHC (g/dL) | 38.3±0.44 | 38.38±0.44 | 0.77 |
| PLT (10^9^/L) | 568.6±38.58 | 579.2±70.44 | 0.78 |

Abbreviations: Hb, hemoglobin; HCT, hematocrit; MCH, mean corpuscular hemoglobin; MCHC, mean corpuscular hemoglobin concentration; MCV, mean corpuscular volume; PLT, platelets; RBC, red blood cell count.

^a^ Student’s *t*-test.

| **Gene** | **Forward primer (5’‒3’)** | **Reverse primer (5’‒3’)** |
| --- | --- | --- |
| **Human genes** | | |
| *HAMP1* | CAGCTGGATGCCCATGTTC | CAGCAGCCGCAGCAGAA |
| *IL-6* | CCCCTGACCCAACCACAAAT | AGCTGCGCAGAATGAGATGA |
| *PTGS2* | CCACCCGCAGTACAGAAAGT | TCTGCCTGCTCTGGTCAATG |
| *β-actin* | CACGGCATCGTCACCAACT | CACGCAGCTCATTGTAGAAGGT |
|  |  |  |
| **Mouse genes** | | |
| *Hamp1* | GCACCACCTATCTCCATCAAC | TTCTTCCCCGTGCAAAGG |
| *Id1* | CGCAGCCACCGGACTCT | AACCCCCTCCCCAAAGTC |
| *IL-6* | TGTATGAACAACGATGATGCACTT | ACTCTGGCTTTGTCTTTCTTGTTATC |
| *IL1-β* | GATGATAACCTGCTGGTGTGTGA | GTTGTTCATCTCGGAGCCTGTAG |
| *Tnf-α* | GACGTGGAACTGGCAGAAGAG | ACCGCCTGGAGTTCTGGAA |
| *Epo* | TCCCCCACGCCTCATCT | TTTCTGCCTCCTTGGCCTCTA |
| *Ptgs2* | CTGCGCCTTTTCAAGGATGG | GGGGATACACCTCTCCACCA |
| *Gpx4* | CCTCCCCAGTACTGCAACAG | GGCTGAGAATTCGTGCATGG |
| *β-actin* | AAATCGTGCGTGACATCAAAGA | GCCATCTCCTGCTCGAAGTC |

**Supplementary** **Table S3. Sequences of primers used for real-time PCR analysis**

**Supplementary Figures**

Supplementary Figure S1

**Supplementary Figure S1. Estrogen reduces the effects of AUR on IL-6 and hepcidin expression.** (a, b) *IL-6* and *HAMP1* mRNA were measured in Huh7 cells treated with 50 ng/ml or the indicated concentration of β-estradiol (E2) for 12 hours. (c-e) *IL-6* and *HAMP1* mRNA levels (c, d), and P-SMAD1/5, SMAD1, P-STAT3, STAT3 and β-ACTIN proteins (e) were measured in Huh7 cells pretreated with E2 (10^-7^ M) for 1 hour, followed by AUR (0.5 μM) for an additional 18 hours. The mRNA levels were normalized to *β-ACTIN* and are expressed relative to the mean of control value. For the protein quantification, P-STAT3 and P-SMAD1/5 were normalized to STAT3 and SMAD1, respectively, and are expressed relative to each of their controls. Error bars indicate the SEM. All data were compared using a one-way ANOVA with Tukey’s *post hoc* test; groups labeled without a common letter were significantly different (*p*<0.05).

Supplementary Figure S2

**Supplementary Figure S2. Iron contents in high-dose AUR treated *Hfe^-/-^* mice.** Starting at 16 weeks of age, male *Hfe^-/-^* mice were received daily intraperitoneal injections of saline (control), AUR (25 mg/kg body weight), or AUR and Fer-1 (1 mg/kg body weight) for 30 days, after which serum iron content and transferrin saturation (a), liver iron content (b), and *Hamp1* mRNA (c) were measured (n=5 mice per group). The mRNA levels were normalized to *β-actin* and are expressed relative to the mean of control value. Error bars indicate the SEM. Data were analyzed using a one-way ANOVA with Tukey’s *post hoc* test; groups labeled without a common letter were significantly different (*p*<0.05).

Supplementary Figure S3

**Supplementary Figure S3. GSH and GPX in high-dose AUR treated *Hfe^-/-^* mice.** Hepatic GSH content (a), *Gpx4* mRNA (b), and total Gpxs activity (c) were measured in male *Hfe^-/-^* mice (n=5 mice per group) following by daily intraperitoneal injections of saline (control), or AUR (25mg/kg body weight) with or without Fer-1 (1mg/kg body weight) for 6 weeks. The mRNA levels were normalized to *β-actin* and are expressed relative to the mean of control value. Error bars indicate the SEM. All data were analyzed using a one-way ANOVA with Tukey’s *post hoc* test, and no significant difference (*p*<0.05) was detected; N.S., no significant difference.

Supplementary Figure S4

**Supplementary Figure S4. TRi-1 treatment induces ferroptosis index in wildtype C57BL/6J mice.** Body weight (a), serum ALT activity (b), hepatic MDA content (c) and hepatic *Ptgs2* mRNA (d) were measured in male wildtype C57BL/6J mice (n=5 mice per group) treated with daily intraperitoneal injections of saline (control), or TRi-1 (25 mg/kg body weight) with or without Fer-1 (1mg/kg body weight) for 2 weeks. The mRNA levels of the indicated genes were normalized to *β-actin* and are expressed relative to the mean of control value. Error bars indicate the SEM. All other data were analyzed using a one-way ANOVA with Tukey’s *post hoc* test; **p*<0.05, groups labeled without a common letter were significantly different (*p*<0.05), and N.S., no significant difference.

Supplementary Figure S5

**Supplementary Figure S5. Auranofin did not increase *IL-6* and *HAMP1* mRNA level in HepG2 cells.** (a) *IL-6* mRNA and (b) *HAMP1* mRNA levels were measured in HepG2 cells treated with the indicated concentration of Auranofin for 6hrs. The mRNA levels were normalized to *β-ACTIN* and expressed relative to the mean value of 0 μΜ group. Error bars indicate the SEM. All data were compared using a one-way ANOVA with Tukey’s *post hoc* test, **p*<0.05, and N.S., no significant difference.

Supplementary Figure S6


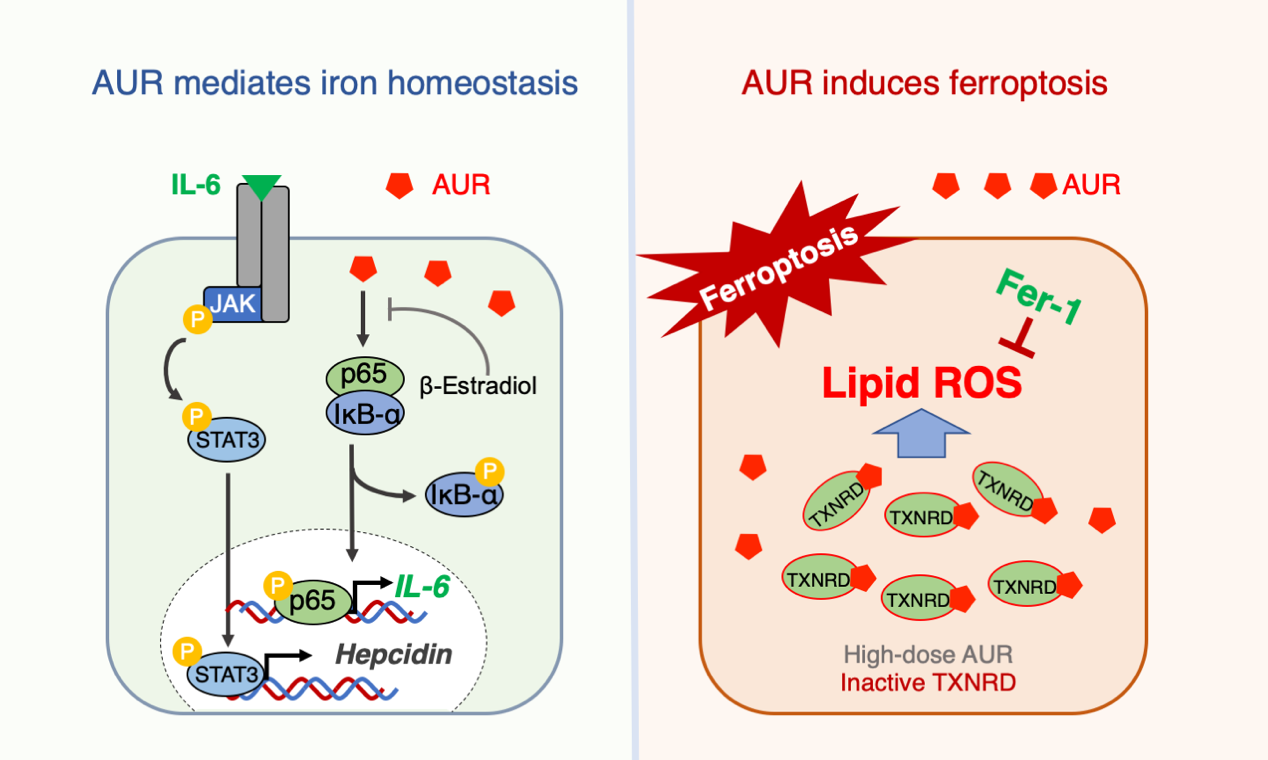


**Supplementary Figure S6. Schematic model depicts the regulatory role of AUR in iron homeostasis and ferroptosis.** Left panel: AUR upregulates hepcidin mRNA expression through NF-κB/IL-6/STAT3 axis, whereas estradiol suppresses AUR-induced hepcidin expression by inhibition of *IL-6* transcription. Right panel: high-dose AUR triggers lipid ROS accumulation and ferroptosis via inactivation of TXNRD. Fer-1 (Ferrostatin-1, ferroptosis inhibitor) scavenges lipid ROS and protects against high-dose AUR-induced ferroptosis.
